# Comprehensive Analyses of Simple Sequence Repeat (SSR) in Bamboo Genomes and Development of SSR Markers with Peroxidase Genes

**DOI:** 10.3390/genes13091518

**Published:** 2022-08-24

**Authors:** Yan Liu, Xiaoyan Xiao, Guangzhu Li, Chenglei Zhu, Kebin Yang, Xiaohu Feng, Yongfeng Lou, Zhimin Gao

**Affiliations:** 1 Institute of Gene Science and Industrialization for Bamboo and Rattan Resources, International Center for Bamboo and Rattan, Beijing 100102, China; 2 Key Laboratory of National Forestry and Grassland Administration/Beijing for Bamboo & Rattan Science and Technology, Beijing 100102, China; 3 Beijing Black Bamboo Park, Beijing 100048, China; 4 Jiangxi Provincial Key Laboratory of Plant Biotechnology, Jiangxi Academy of Forestry, Nanchang 330013, China

**Keywords:** bamboo, SSR, whole-genome identification, comparative analyses, molecular markers

## Abstract

Simple sequence repeats (SSRs) are one of the most important molecular markers, which are widespread in plants. Bamboos are important forest resources worldwide. Here, the comprehensive identification and comparative analysis of SSRs were performed in three woody and two herbaceous bamboo species. Altogether 567,175 perfect SSRs and 71,141 compound SSRs were identified from 5737.8 Mb genome sequences of five bamboo species. Di-nucleotide SSRs were the most predominant type, with an average of ~50,152.2 per species. Most SSRs were located in intergenic regions, while those located in genic regions were relatively less. Moreover, the results of annotation distribution indicated that terms with P450, peroxidase and ATP-binding cassette transporter related to lignin biosynthesis might play important roles in woody and herbaceous bamboos under the mediation of SSRs. Furthermore, the peroxidase gene family consisted of a large number of genes containing SSRs was selected for the evolutionary relationship analysis and SSR markers development. Fifteen SSR markers derived from peroxidase family genes of *Phyllostachys edulis* were identified as polymorphic in 34 accessions belonging to seven genera in Bambusoideae. These results provided a comprehensive insight of SSR markers into bamboo genomes, which would facilitate bamboo research related to comparative genomics, evolution and marker-assisted selection.

## 1. Introduction

Molecular markers usually refer to specific DNA segments that can reflect certain differences in the genomes of individuals or populations, which can directly reveal the genetic information in the organism through DNA molecules [1]. It is widely used in cultivar identification, marker-assisted selection, and genetic diversity analysis of germplasm resources [2]. Based on the method of analyses, molecular markers can be divided into three classes: (1) non-PCR-based techniques but based on hybridization, such as restriction fragment length polymorphism (RFLP); (2) PCR-based techniques, such as random amplified polymorphic DNA (RAPD), amplified fragment length polymorphism (AFLP), and simple sequences repeat (SSR); (3) sequence-based marker techniques that is single nucleotide polymorphism (SNP) [1,3]. In comparison to other molecular markers, SSR markers characterized by high variable, co-dominance and high polymorphism, are deemed as one of the most powerful genetic markers [3]. SSR identification is mainly relied on in the construction and screening of SSR-enriched libraries in earlier studies [4,5]. In recent years, with the rapid development of next-generation sequencing technology, genome-wide SSR identification has been conducted in plants such as *Brassica oleracea* [6], *Psidium guajava* [7] and *Camellia sinensis* [8], which have been considered as a more effective and comprehensive method [3]. Moreover, many studies suggest that SSRs in genic regions might affect gene regulation, translation, gene silencing, messenger RNA splicing, and metabolic activities [9,10,11].

Bamboos belong to Bambusoideae consisting of 75 genera accommodating 1642 bamboo species [12], which are classified into three tribes: Arundinarieae (temperate woody bamboos), Bambuseae (tropical woody bamboos), and Olyreae (herbaceous bamboos) [13]. Bamboos are important non-timber forest resources. Molecular markers like RAPD, AFLP, SSR, and SNP were applied to bamboo species classification and genetic diversity analyses [12,14]. Several SSR markers of bamboo were developed to analyze the genetic diversity and phylogenetic of bamboo, which was helpful for the genetic improvement and taxonomic studies of bamboo [12,15]. However, previous studies demonstrated that the number of bamboo SSR markers identified with polymorphism was limited, due to the lack of a large sequence data [12]. To date, the genome-wide analysis of SSR identification was only performed with the draft genome of *P. edulis*, and 20 SSR markers were developed by random location selection to distinguish 78 bamboo accessions belonging to *Phyllostachys* [16]. Nonetheless, compared with SSR markers derived from random genomic locations, SSR markers derived from genes would be likely to provide much more genetic information because they represented the variations in gene coding or regulatory region [17]. Besides, with the release of the chromosome-level reference genome of *P. edulis* and the draft genomes of *Olyra latifolia*, *Raddia guianensis*, *Guadua angustifolia* and *Bonia amplexicaulis* [18], comparative analysis of SSR identification at whole-genome level between *P. edulis* and related bamboo species was insufficient. Thus, it is necessary to identify the SSRs at whole-genome level for full understanding of their characteristics and potential biological functions in bamboos.

Woody bamboo is environmentally friendly, abundant, and an alternative to conventional timber [19]. Wood formation distinguishes woody from herbaceous plants, and represents a major metabolic sink for woody plants [17]. Several polymorphic SSR markers identified within genes associated with woody trait were helpful for marker-assisted breeding [17,20,21]. Lignin is the main component of secondary cell walls, and is essential for wood formation [22,23]. Lignin biosynthesis is completed by a series of enzymes, such as phenylalanine ammonia lyase, cinnamate 4-hdroxylase and peroxidase [24]. In this study, the characteristics of SSRs were analyzed and compared based on the released genome sequences of five bamboo species representing three tribes: two herbaceous species, *O. latifolia* and *R. guianensis*, belonging to Olyreae; two woody species, *G. angustifolia* and *B. amplexicaulis*, belonging to Bambuseae; and one woody species, *P. edulis*, belonging to Arundinarieae [12,13,18]. Moreover, the potential functions of genes containing SSR were further statistically investigated. Furthermore, peroxidase gene family with a large number of genes containing SSRs was analyzed, in which SSR markers were developed. As a result, 15 pairs of SSR markers derived from peroxidase family genes of *P. edulis* were identified as polymorphic in 34 accessions belonging to seven genera in Bambusoideae. This research provides potentially important molecular markers for the study of population genetics, fingerprinting, and regulatory mechanisms of functional genes in bamboo species.

## 2. Materials and Methods

### 2.1. Sequence Retrieval and SSR Identification

All SSRs were identified from five bamboo genomes (*P. edulis* data from BambooGDB [25], other four of *B. amplexicaulis*, *G. angustifolia*, *O. latifolia*, and *R. guianensis* from Bamboo genome database [18]) using the microsatellite identification tool (MISA) [26,27] with the following parameters: minimum number of 12 repeats for mononucleotide motif, 6 repeats for dinucleotide motif, 5 repeats for trinucleotide motif, 4 repeats for tetra-, penta-, and hexanucleotide motifs, 3 repeats for hepta-, octa-, nona-, and decanucleotide motifs. The maximum distance between two SSRs in a compound sequence was less than 100 bp.

### 2.2. Primer Design for SSR Markers

Primers were designed for all identified SSRs using the Primer3 [28]. The main parameters were set as follows: (i) the primer length ranged from 18 bp to 27 bp, (ii) the annealing temperature ranged from 57 °C to 65 °C, (iii) the size of target PCR products ranged from 100 bp to 300 bp, (iv) the GC content ranged from 40% to 60%. All other parameters were set to the default values according to the Primer3 program.

### 2.3. Functional Annotation and Identification of Peroxidase Family Genes

The functional annotation of all genes in five bamboo species was conducted using Pfam database [29]. Proteins containing ‘peroxidase’ (PF00141) domain were extracted using a customized Python program. The protein sequences of all genes in five bamboo species were aligned with Swiss-Prot database [30] using Blast software (v2.10.0+, https://blast.ncbi.nlm.nih.gov/Blast.cgi) with an e-value < 1 × 10^−5^. The successful annotated peroxidase genes were screened using a customized Python program based on the following criteria: (i) the percentage of identical matches was more than 40, (ii) the alignment length was more than 80 aa, (iii) the ‘peroxidase’ was contained in the subject title.

### 2.4. Evolution Analysis of Peroxidase Gene Family

The protein sequences of peroxidase family members were aligned using the Mafft software (v7.487, https://mafft.cbrc.jp/alignment/software/) [31]. A phylogenetic tree was constructed using the FastTree software (v2.1.11, http://www.microbesonline.org/fasttree/) [32] with 1000 bootstrap replications. The Jones–Taylor–Thorton (JTT) model was used for phylogenetic analysis. The phylogenetic trees of the peroxidase family genes were constructed using the iTOL program (https://itol.embl.de/) [33]. The paralogous and orthologous relationships between the peroxidase genes of *P. edulis* and others bamboo species were identified using Orthofinder software (v2.4.0, https://github.com/davidemms/OrthoFinder) [34]. Images of the statistical distribution of orthologous peroxidase gene pairs between *P. edulis* and the other four bamboo species were drawn using Circos software (v0.69-8, http://circos.ca/) [35]. 

### 2.5. Ka/Ks Calculation and Divergent Time Prediction

To estimate the divergence of the paralogous and orthologous genes, the protein sequences of paralogous peroxidase family gene pairs in *P. edulis* and orthologous peroxidase family gene pairs between *P. edulis* and the other four bamboo species were aligned using the Mafft software (v7.487, https://mafft.cbrc.jp/alignment/software/) [31]. Then, the value of the non-synonymous (*Ka*), synonymous (*Ks*) and *Ka*/*Ks* of those gene pairs were calculated according to their coding sequence alignment by using Nei-Gojobori method implemented in the KaKs_calculator program (v2.0, https://github.com/kullrich/kakscalculator2) [36]. The divergence time was estimated based on the substitution rate 6.5 × 10^−9^ substitutions per site per year [37].

### 2.6. Plant Materials, Genomic DNA Isolation, and Detection

Leaf samples of the accessions belonging to seven genera of Bambusoideae were collected from the Black Bamboo Park, Beijing, and the base of Jiangxi Academy of Forestry Sciences, Nanchang City, in Jiangxi Province, respectively. The accessions are shown in Supplementary Table S1. Genomic DNA (gDNA) of leaves was extracted by a modified CTAB method, originally described by Doyle and Doyle [38]. The quality and quantity of DNA were detected using Nanodrop 2000. 

### 2.7. SSR Polymorphism Assessment

The gDNA of *P. edulis* was selected as template to validate the suitability of 48 SSR primers pairs identified from peroxidase genes in *P. edulis*. Standard PCR was carried out in a reaction volume of 20 μL using 2× Taq Mix (Vazyme, Nanjing, China) according to the manufacturer’s instructions. PCR amplification was performed by thermocycler gradient using the following profile: 95 °C for 3 min, followed by 35 cycles of 95 °C for 25 s, appropriate annealing temperature for 25 s, 72 °C for 18 s, and a final extension at 72 °C for 5 min. Amplified products were electrophoresed in 2.0% agarose gel.

TP-M13-SSR PCR method was used to testify the polymorphism of the selected SSR primers among 34 bamboo accessions. This method contained a forward primer with a universal M13 primer tail (5′-TGTAAAACGACGGCCAGT-3′) at the 5′ end and a universal M13 primer fluorescently labeled with 6-carboxy-x-rhodamine, 6-carboxy-fluorescein, tetramethyl-6-carboxyrhodamine, or 5-hexachlorofluorescein [39]. The PCR conditions were as follows: 95 °C for 3 min, followed by 20 cycles of 95 °C for 25 s, appropriate annealing temperature for 25 s, 72 °C for 18 s, 95 °C for 25 s, 52 °C for 25 s, 72 °C for 18 s, and a final extension at 72 °C for 5 min. The final products were used for SSR analysis based on capillary electrophoresis fluorescence on the ABI 3730 DNA Analyzer (Rui Biotech Inc., Beijing, China). The results were analyzed using GeneMarker software (v2.2.0, https://softgenetics.com/products/genemarker/) [40]. Unweighted Pair Group Method with Arithmetic Mean (UPGMA) cluster analysis was performed for 34 bamboo accessions using PowerMarker (v3.25, https://brcwebportal.cos.ncsu.edu/powermarker/) [41].

## 3. Results

### 3.1. Comprehensive SSR Identification

A total of 638,631 SSRs containing repeats from mono- to deca-nucleotides were identified from 5737.8 Mb of the whole genome sequences in five bamboo species, with an average density of 111.2 SSRs/Mb (Table 1). The results demonstrated the highest SSRs frequency was found in herbaceous species *R. guianensis* (157.3 SSRs/Mb) whose genome was minor. Moreover, among the five genomes analyzed, the biggest genome size of *P. edulis* (1907.6 Mb) was approximately 3.0 times that of *R. guianensis* (626.4 Mb), and the SSR numbers in *P. edulis* (185,102) were about twice those in *R. guianensis* (98,511), whereas the frequency of SSR in *R. guianensis* (157.3 SSRs/Mb) was ~1.6-fold that in *P. edulis* (97.0 SSRs/Mb).

**Figure 1:**
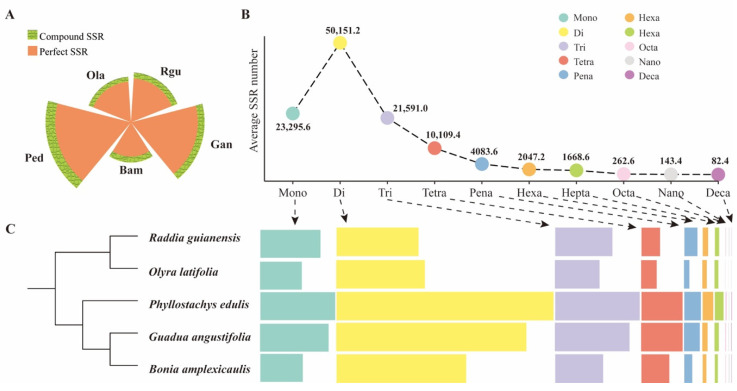
Comparison of the distribution of different SSR types in five bamboo species of *B. amplexicaulis* (Bam), *G. angustifolia* (Gan), *P. edulis* (Ped), *O. latifolia* (Ola), and *R. guianensis* (Rgu). (**A**) The distribution of perfect SSRs and compound SSRs in different bamboo species. (**B**) The average number of ten SSR types (mono- to decanucleotides) in five bamboo species. (**C**) The boxplots indicating the SSR number of each type in different species.

Perfect SSRs are defined as continuous repetitions without any interruption [3], while the others were classified as compound SSRs. In this study, 567,175 perfect SSR motifs and 71,141 compound SSR motifs were identified from five bamboo genomes. Perfect SSRs were the dominant type in different bamboo species, ranging from 85.2% (*O. latifolia*) to 91.0% (*G. angustifolia*) (Figure 1A and Supplementary Table S2). According to their repeat compositions, all the identified perfect SSRs were classified (Figure 1B,C). The dinucleotide repeat was the most abundant, with an average number of ~50,151.2 per species, whereas the decanucleotide was rarely detected in the examined sequences, with an average number of ~82.4 per species. Further analysis showed that *P. edulis* owned the largest number in various types of repeat compositions except for tetra-nucleotide repeat, which was predominant in *G. angustifolia*.

### 3.2. Comparison of SSR Characteristics in Different Bamboo Species

**Figure 2:**
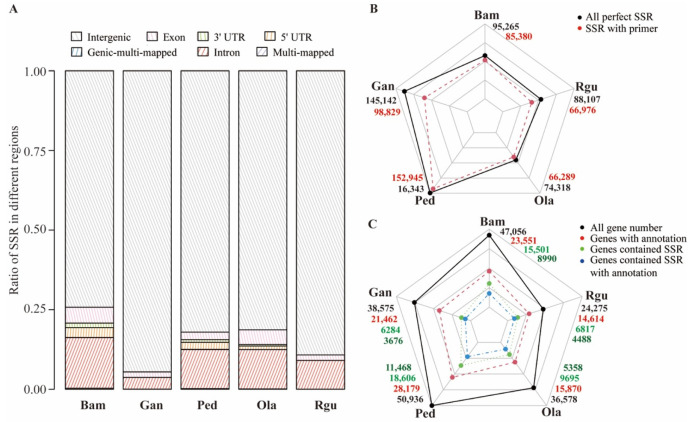
Comparison of the SSR characteristics among five bamboo species of *B. amplexicaulis* (Bam), *G. angustifolia* (Gan), *P. edulis* (Ped), *O. latifolia* (Ola), and *R. guianensis* (Rgu). (**A**) The distribution of SSRs located in different regions of genome in each bamboo species. (**B**) The total number of perfect SSRs and the number of perfect SSRs with successfully designed primers in each bamboo species. (**C**) The total number of genes, the total number of genes with Pfam annotations, the number of genes containing SSR, and the number of genes containing SSR with Pfam annotations in each bamboo species.

SSRs located in different genomic regions may perform various functions [9]. According to the location and annotation of genes, the genome sequences were classified into seven regions. Besides intergenic, exon, intron, 5′ UTR (Untranslated Regions), and 3′ UTR regions, several SSRs located into multiple regions were classified as multi-mapped or genic-multi-mapped regions. For instance, SSRs that aligned with both intergenic and genic regions were classified into multi-mapped regions, and SSRs that were able to match two or more genic regions were classified into genic-multi-mapped regions. Moreover, none of SSRs belonging to 5′ UTR and 3′ UTR regions were detected in *G. angustifolia* and *R. guianensis* because of these regions’ lack of annotation. The results showed most SSRs were commonly mapped onto intergenic regions, followed by genic regions, the least onto multi-mapped regions (Figure 2A and Supplementary Table S3). More than 25% SSRs were located in genic regions in *B. amplexicaulis*. *O. latifolia*, *P. edulis* and *B. amplexicaulis* were further selected to compare the SSR distribution differences in different genic regions (Figure 2A and Supplementary Table S4). The results illustrated that most of the SSRs were located in intron, followed by exon, 5′ UTR and 3′ UTR, and the least were located in genic-multi-mapped regions. *P. edulis* possessed the largest number of SSRs located in various genic regions except exon, which was predominant by *B. amplexicaulis*. Primers were designed for all perfect SSRs. A total of 470,419 primer pairs (for 82.9% of total perfect SSR motifs) were generated from the 567,175 perfect SSRs in examined five bamboos genomes in this study (Figure 2B and Supplementary Table S5). Among them, *P. edulis* showed the highest percentage of successfully designed primers for 93.1%. By contrast, merely 68.1% SSRs in *G. angustifolia* had successful primers.

The gene number of *P. edulis* (50,936) was the highest, which was ~2 times that of *R. guianensis* (24,275), and the number of SSR-containing genes (18,606) was ~3 times that of *G. angustifolia* (6284) (Figure 2C). To further investigate the function of genes containing SSRs, Pfam database was used to conduct functional annotation. *P. edulis* had the highest total number of annotated genes (28,179), followed by *B. amplexicaulis* (23,551) (Figure 2C). Merely 14,614 genes were annotated in *R. guianensis* genome, which owned the lowest one. It was obvious that the total number of gene annotations was highly related to the number of genes containing SSR (r = 0.99). *P. edulis* had the highest percentage of annotated SSR-containing genes (40.7%) among five bamboos, while *G. angustifolia* was the lowest with that of 17.1%.

### 3.3. Statistical Analysis of the SSR-Containing Gene Functions

**Figure 3:**
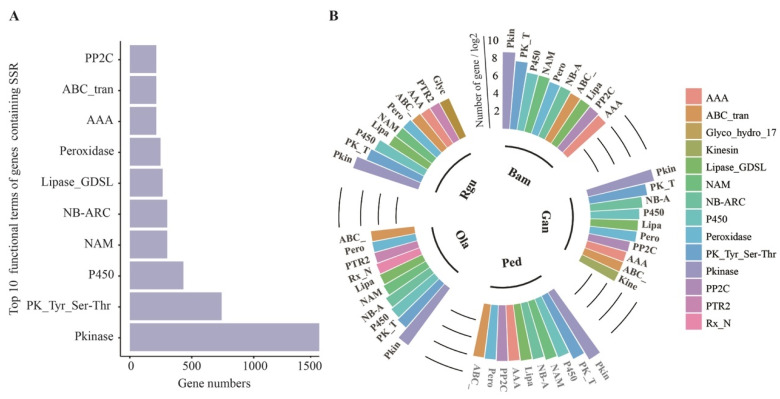
Statistics analysis of functional Pfam terms of SSR-containing genes in five bamboo species of *B. amplexicaulis* (Bam), *G. angustifolia* (Gan), *P. edulis* (Ped), *O. latofolia* (Ola), and *R. guianensis* (Rgu). (**A**) The top 10 terms of total SSR-containing genes in five bamboo species. (**B**) The top 10 terms of SSR-containing genes in each bamboo species.

Altogether, 33,980 genes containing SSRs were annotated in Pfam database. Then, the statistical distribution of the top 10 Pfam terms was analyzed (Figure 3A). Among them, the highest number of SSR-containing genes annotated was Pkinase (Protein kinase domain), followed by PK_Tyr_Ser-Thr (Protein tyrosine and serine/threonine kinase), and P450 (Cytochrome P450). Interestingly, among the top 10 Pfam terms, P450 was related to lignin biosynthesis [42], NAM (No apical meristem) was involved in regulating lignin biosynthesis [24], ABC_tran (ATP-binding cassette transporter) was lignin monomer transporter [43], and peroxidase was involved in lignin monomer polymerization [44]. The results indicated that SSRs might be related to lignin biosynthesis and regulation. The genes containing SSR annotated Pfam terms in each bamboo species were ranked by the number as well (Figure 3B). The results showed that Kinesin (Kinesin motor domain), Glyco_hydro_17 (Glycoside hydrolase family 17), and Rx_N (Rx N-terminal domain) were specific to *G. angustifolia*, *R. guianensis*, and *O. latifolia*, respectively. Pkinase, PK_Tyr_Ser-Thr, P450, Lipase_GDSL (GDSL-like Lipase/Acylhydrolase), peroxidase, and ABC_tran were shared in the top 10 Pfam terms both in woody and herbaceous bamboos. These results suggested that genes containing P450, peroxidase and ABC_tran related to lignin biosynthesis might play important roles mediated by SSRs in woody and herbaceous bamboos.

### 3.4. Analysis of the Peroxidase Gene Family

The above analyses showed that most SSRs were located in the genes annotated with peroxidase in each bamboo species. Thus, the evolutionary relationships of peroxidase family genes between *P. edulis* and the other four bamboo species were further analyzed. In the genomes of five bamboo species, altogether 923 genes were identified as members of peroxidase family according to both Pfam annotation and Swiss-Prot annotation (Supplementary Table S4). Compared with the other four bamboo species, the number of peroxidase genes in *P. edulis* (226) was the highest, almost identical to that of *G. angustifolia* (224) but ~1.8 times that of *R. guianensis* (124). A total of 240 SSR-containing peroxidase genes were identified in five bamboo species (Supplementary Table S6), accounting for 26.0% of all identified peroxidase genes. *B. amplexicaulis* (34.5%) showed the highest ratio of peroxidase genes containing SSR, followed by *P. edulis* (29.6%) and *R. guianensis* (27.4%).

**Figure 4:**
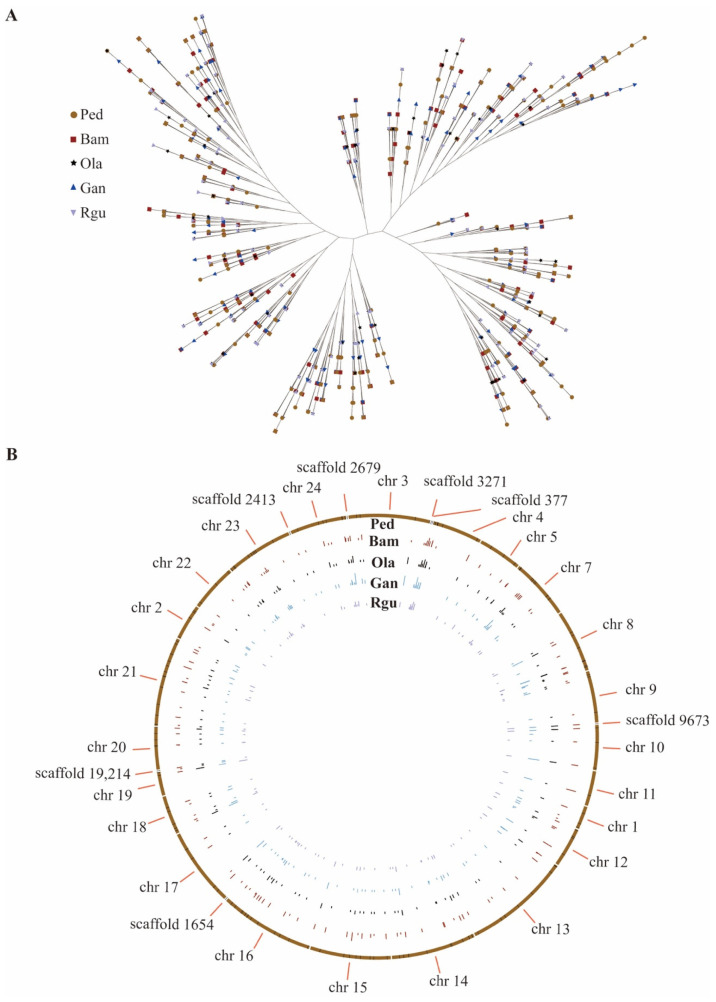
Phylogenetic relationship and chromosome distribution analyses of orthologous peroxidase gene pairs between *P. edulis* (Ped) and the other four bamboo species of *B. amplexicaulis* (Bam), *G. angustifolia* (Gan), *O. latifolia* (Ola), and *R. guianensis* (Rgu). (**A**) Phylogenetic tree constructed using the peroxidase from five bamboo species. (**B**) Statistical chromosome distribution of orthologous peroxidase gene pairs between *P. edulis* and the other four bamboo species respectively.

To investigate the evolutionary relationship of peroxidase family, a phylogenetic tree was constructed using a total of 923 amino acid sequences of peroxidase from five bamboo species (Figure 4A). According to the topology of phylogenetic tree, 923 peroxidase family members were divided into five groups. The Orthofinder software was used to analyze the paralogous of peroxidase genes in *P. edulis*, which had the largest number of peroxidase genes in five bamboo species. The results showed that there were 119 paralogous gene pairs identified in *P. edulis*. Then the orthologous analyses of peroxidase genes among *P. edulis* and the other four bamboo species were performed using the Orthofinder software as well. A total of 806 orthologous gene pairs were identified between *P. edulis* and the other four bamboo species. Among them, *P. edulis* and *G. angustifolia* had the largest number of orthologous gene pairs (253), followed by *P. edulis* and *B. amplexicaulis* with 221 pairs (Figure 4B and Supplementary Table S7).

**Figure 5:**
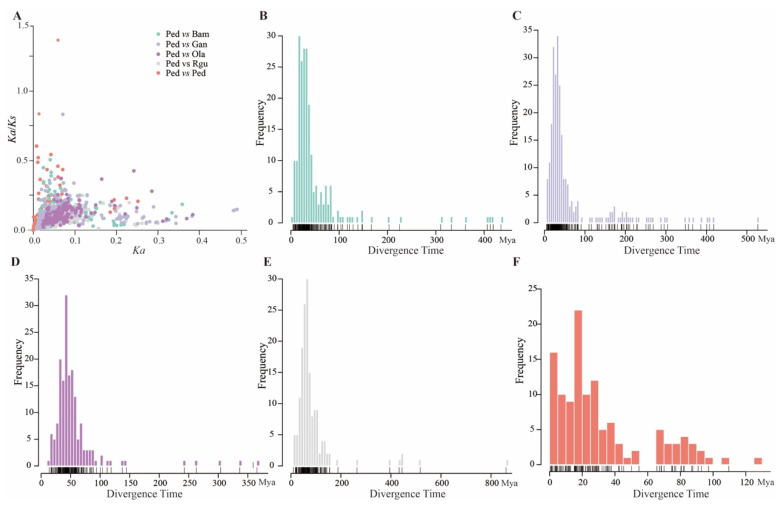
Evolutionary analyses of paralogous peroxidase gene pairs of *P. edulis* and orthologous peroxidase gene pairs between *P. edulis* (Ped) and the other four bamboo species of *B. amplexicaulis* (Bam), *G. angustifolia* (Gan), *O. latifolia* (Ola), and *R. guianensis* (Rgu). (**A**) The *Ka*/*Ks* values of paralogous peroxidase gene pairs in *P. edulis* and orthologous peroxidase gene pairs between *P. edulis* and the other four bamboo species. (**B**) The divergence time of orthologous peroxidase gene pairs between *P. edulis* and *B. amplexicaulis*. (**C**) The divergence time of orthologous peroxidase gene pairs between *P. edulis* and *G. angustifolia*. (**D**) The divergence time of orthologous peroxidase gene pairs between *P. edulis* and *O. latifolia*. (**E**) The divergence time of orthologous peroxidase gene pairs between *P. edulis* and *R. guianensis*. (**F**) The divergence time of paralogous peroxidase gene pairs in *P. edulis*.

In addition, the selection types and divergence time of the analyzed orthologous and paralogous peroxidase gene pairs were calculated according to the nonsynonymous substitutions (*Ka*) and synonymous substitutions (*Ks*). The results showed that *Ka*/*Ks* ratio of all (806) orthologous gene pairs was less than 1, and *Ka*/*Ks* ratios of the most (280/281) paralogous gene pairs were less than 1 as well (Figure 5A), indicating that they had undergone purifying selection during evolution. Furthermore, the divergence time was calculated according to a synonymous substitution rate. To visualize the data, the distributions of the divergence time of paralogous peroxidase gene pairs in *P. edulis* and orthologous peroxidase gene pairs between *P. edulis* and the other four examined bamboo species in units of 5 million years ago (Mya) were calculated (Figure 5B–F). The results indicated that the divergence time of orthologous peroxidase gene pairs between *P. edulis* and *R. guianensis* was concentrated on 50–65 Mya, and that between *P. edulis* and *O. latifolia* was focused on 30–40 Mya. The concentration divergence time of orthologous peroxidase gene pairs between *P. edulis* and *B. amplexicaulis* (15–30 Mya) was nearly identical to that between *P. edulis* and *G. angustifolia* (20–30 Mya). The divergence time of paralogous peroxidase gene pairs in *P. edulis* was within 15 Mya.

### 3.5. Development SSR Primer Pairs in Peroxidase Genes

**Figure 6:**
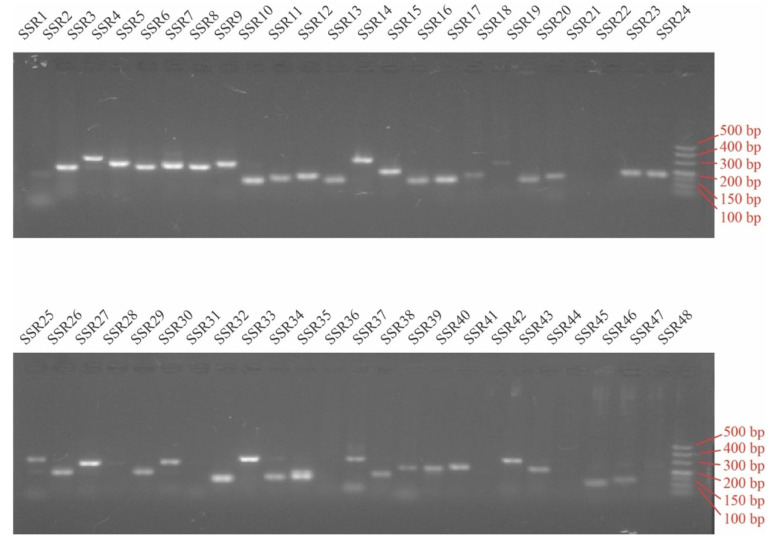
Amplification of 48 SSR primers using genomic DNA of *P. edulis* as template.

Nowadays, few researchers employ mononucleotide repeat-motif as molecular markers because of their instability [16]. Thus, to validate the polymorphism of SSR primers in peroxidase genes, 48 (60.0%) pairs of SSR primers were selected and synthesized, which were designed based on the SSR-containing peroxidase genes (80) in *P. edulis* except mono-nucleotide SSRs (Supplementary Tables S8 and S9). Of these, 40 primer pairs produced clear and stable bands with the expected size (Figure 6). Moreover, retrieved from SSR primers validation, 15 primer pairs with high amplification effect were identified as polymorphism for 34 bamboo accessions belonging to seven genera. A total of 89 alleles were detected across 15 SSR loci with an average of 5.93 alleles per SSR locus for 34 samples (Table 2). The maximum genotype number was 20 observed in locus SSR13 (Supplementary Figure S1) followed by 13 detected in locus SSR43 (Supplementary Figure S2). The polymorphism information content (PIC) at each locus ranged from 0.03 to 0.87, with an average of 0.46. Out of 15 markers, seven were highly polymorphic (PIC ≥ 0.50). These results indicated that the SSR markers used were suitable for genetic diversity studies.

**Figure 7:**
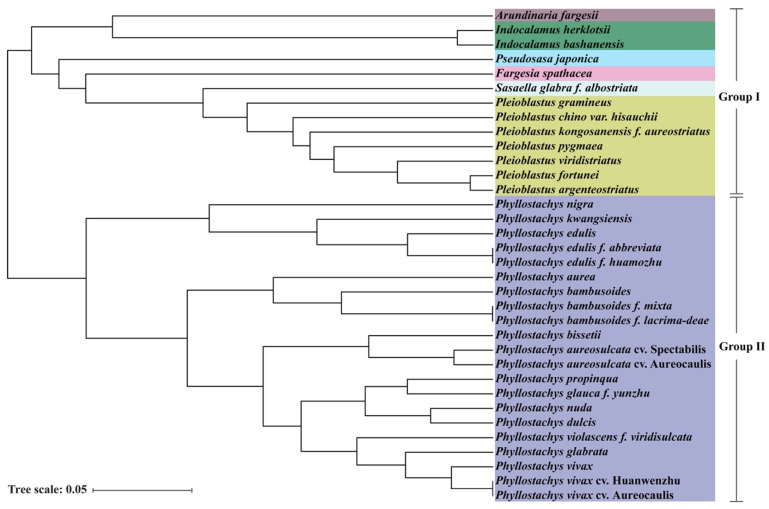
Cluster analysis of 34 bamboo accessions in seven genera based on SSR data. Different colors represent different genera.

Unweighted Pair Group Method with Arithmetic Mean (UPGMA) analysis of SSR data showed that 34 bamboo accessions were clustered into two groups belonging to Arundinarieae (Group I) and Shibataeeae (Group II) respectively (Figure 7). The different genera were clustered separately. Those accessions belonging to the same genera were clustered together, such as two accessions belonging to *Indocalamus* and seven accessions belonging to *Pleioblastus*. Moreover, the cultivars, variants or forma from *P. edulis*, *P. bambusoides*, *P. bambusoides* and *P. vivax* were clustered in the same clade respectively. These clustering results indicated that the majority of the accessions were consistent with their current taxonomic classification. However, the cultivars and forma could not be distinguished using these primer sets, indicating that although the SSR markers of peroxidase genes had polymorphic, it was still limited. The original variant and cultivars or forma could be distinguished, whereas most accession within cultivars or forma could not be distinguished except two accessions of *P. aureosulcata*. For example, *P. edulis* and *P. kwangsiensis* belonging to *Phyllostachys* genus were clustered into different clades of Group II. However, it could not distinguish the two kinds of forma, *P. edulis f. abbreviata* and *P. edulis f. huamozhu*, *P. bambussoides f. mixta* and *P. bambusoides f. lacrima-deae*, as well as the cultivars *P. vivax* cv. Huangwenzhu and *P. vivax* cv. Auroecaulis.

## 4. Discussion

SSRs have been deemed as promising candidate markers for population genetics and germplasm identification in plants due to their hypervariability, high information content and codominance [45,46,47]. With the rapid development of next-generation sequencing technology, whole-genome SSR identification becomes much more effective and comprehensive [2,48]. In this study, whole-genome SSR identification and comparative analysis were carried out in five bamboo species including two herbaceous bamboos and three woody bamboos representing three tribes. A total of 638,316 SSRs were identified from 5737.8 Mb sequences in five bamboo genomes. The results seemed to be consistent with the previous study that species possessing larger genomes showed a larger number of SSRs, but lower SSR density [49,50]. Nonetheless, *P. edulis* possessing the largest genome size did not display the lowest density of SSR, and *R. guianensis* with the smallest genome size did not show the minimum number of SSR as well (Table 1). Thus, the SSR density and SSR number may be not associated with genome size.

Moreover, the distribution of SSR repeat motifs was similar in five examined bamboo species. For instance, the repeat motifs within tri-nucleotide were the dominant types (Figure 1), which was in accordance with the previous study found in whole-genome SSR identification in the draft genome of *P. edulis* [16]. In addition, two woody bamboo species *P. edulis* and *G. angustifolia* showed more SSR numbers in various SSR repeat motifs. Generally, most SSRs were located in the intergenic region, and the ratio of SSRs located in the coding sequences was relatively small [4]. Our study had a similar result (Figure 2). In addition, SSR distribution is the result of selection pressure during the evolutionary process [51]. Developing SSR markers derived from functional genes will likely provide a much greater degree of resolution in marker assisted selection and association mapping [17]. Here, a total of 56,903 with a range of 6284 (*G. angustifolia*) to 18,606 (*P. edulis*) genes containing SSR loci were detected in five surveyed bamboo species. The statistical analysis of the top 10 Pfam annotation terms of SSR-containing genes showed several terms were found to be related to lignin, indicating many SSR markers were widely present in genes related to lignin biosynthesis or regulation (Figure 3). For instance, genes containing P450 or peroxidase domain may take part in lignin biosynthesis, and G-type ATP-bind cassette (ABCG) transporters containing ABC transporter domain may be involved in monolignol transporter [43]. NAC (NAM, ATAF and CUC) and MYB transcription factors are the main compositions of the lignin biosynthesis regulatory networks [19,24]. Many genes involved in lignin biosynthesis or regulation contained SSR loci, which might lead to the difference in lignin content among different bamboo species, thus dividing them into herbaceous and woody bamboo species. Therefore, this provided a reliable resource for the process of marker-assisted selection in bamboo.

Genes encoding peroxidases play key roles in several important physiological and developmental processes, such as lignin and suberin formation, crosslinking of cell wall components, and defense against pathogens [44,52,53,54]. In the present study, 923 peroxidase genes were identified from the whole genome of five bamboo species. The number of 227 peroxidase genes in *P. edulis* was more than two times of previous study [19]. One reason for this may be the different identification methods. Here, the genes annotated with ‘peroxidase’ were filtered in Pfam database and Swiss-Prot database simultaneously. Whereas the peroxidase protein sequences of *Arabidopsis thaliana* or *Oryza sativa* were firstly downloaded in the previous study and then compared with the protein sequences of *P. edulis* by BLAST, the sequences lacking complete peroxidase domain were filtered out finally using Pfam database and SMART. Another reason for the number gap was that the identified genes involved in lignin biosynthesis belonged to Class III peroxidases [55,56]. In fact, higher plants contained at least four types of peroxidases including classical secretory plant peroxidases (Class III peroxidase) [55]. Based on the systematic comparative analyses and phylogenetic analyses (Figure 4 and Figure 5), the divergence time of paralogous peroxidase gene pairs in *P. edulis* was within 15 Mya, which was consistent with the whole-genome duplication of *P. edulis* underwent during 7–12 Mya [37]. However, the divergence time between *P. edulis* and the other four examined bamboo species was much earlier and broader than that in the previous study, which was more accurate and comprehensive using 61 ‘perfect-copy’ syntenic genes derived from *O. latifolia*, *B. amplexicaulis*, *G. angustifolia* and *P. edulis* [18].

Generally, the repeat motifs with more than mononucleotide were used for developing molecular markers. Several SSR markers were developed and used for genetic diversity and fingerprinting analyses within some species [57,58]. In addition, genic SSRs exhibited relatively high transferability and availability to closely related species [17,59]. In this study, SSR markers extracted from peroxidase genes of *P. edulis* were highly conservative and transferable from *P. edulis* to some related species in Bambusoideae. The results were consistent with the research on cabbage and popular [6,17]. Meanwhile, a total of 89 alleles with a range of 2 to 16 loci were detected (Table 2), indicating the wide range of diversity among the bamboo accessions. The number of alleles of SSR markers derived from *P. edulis* was relatively smaller in the previous studies [15,16]. For example, the 64 alleles ranging from 2 to 5 per loci were detected by 23 SSR primers, with an average value of 2.78 for 78 accessions belonging to the genus *Phyllostachys*. The differences in the number of alleles were due to the use of different genetic materials [16]. 

Moreover, UPGMA analysis showed accessions belonging to the same or different genera could be clustered into different clades, but the cultivars and forma could not be distinguished (Figure 7), suggesting that species belonged to the same or different genera had more variation in peroxidase genes. For example, *P. edulis* and its two forma could be divided into different clades, whereas it was difficult to distinguish the two forma with the SSR markers. However, the original variant and six forma of *P. edulis* could be distinguished in the previous study [16]. The main reason for the difference of resolution capability between two sets of primers was the different sources. Twenty-three SSRs primers which were filtered from 917 pairs of SSRs, which located random genomic of *P. edulis*, were more likely to distinguish the forma or cultivars compared with the 15 SSRs only derived from peroxidase genes of *P. edulis*. Nevertheless, it was time-consuming and expensive for developing SSR polymorphism markers located in random genomic regions. Alternatively, developing SSR markers originated from functional genes had greater efficiency for species identification, and were beneficial for marker-assisted selection as well.

## 5. Conclusions

A total of 567,175 perfect SSRs and 71,141 compound SSR motifs were identified from 5737.8 Mb sequences of three woody bamboo and two herbaceous bamboo species representing three tribes of Bambusoideae. The dinucleotide SSRs were the predominant type, with an average of ~50,152.2 per species. The number of SSRs located in genic regions was relatively smaller than that of intergenic regions but provided much more genetic information. Out of the top 10 Pfam terms of annotated SSR-containing genes, P450, NAM, ABC transporter and peroxidase related to lignin biosynthesis or regulation were abundant. Furthermore, 15 SSR markers originated from peroxidase genes in *P. edulis* were developed to detect polymorphism in 34 accessions belonging to seven genera in Bambusoideae. Taken together, these results have considerable potential value in advancing bamboo research, including comparative genome analyses, phylogenetic evolution, germplasm identification, classification and molecular marker assisted selection for breeding.

## Figures and Tables

**Table 1:** A comparative survey of SSR identification across five examined bamboo species.

Species	Type	Genome Size (Mb)	SSR Number	Density (SSRs/Mb)
*B. amplexicaulis*	Woody	848.6	107,976	127.2
*G. angustifolia*	Woody	1708.6	159,530	93.4
*P. edulis*	Woody	1907.6	185,102	97.0
*O. latifolia*	Herbaceous	646.6	87,197	134.9
*R. guianensis*	Herbaceous	626.4	98,511	157.3
Total		5737.8	638,316	111.2

**Table 2:** Genetic diversity analysis with polymorphism SSR markers developed in this study.

Primer ID	Genes	Motif	Position	Genotype Number	Na	PIC
SSR2	*PH02Gene25200*	(TAT)5	Intron	2	2	0.03
SSR3	*PH02Gene00439*	(TACA)5	Intron	12	7	0.72
SSR6	*PH02Gene02739*	(AG)6	5′ UTR	2	3	0.40
SSR7	*PH02Gene03921*	(GC)6	Intron	9	8	0.58
SSR8	*PH02Gene05936*	(AGCAG)4	5′ UTR	8	7	0.63
SSR11	*PH02Gene12228*	(CT)7	Intron	12	9	0.75
SSR13	*PH02Gene18893*	(TG)21	3′ UTR	20	16	0.87
SSR14	*PH02Gene21597*	(TGAT)4	Intron	5	3	0.37
SSR17	*PH02Gene25407*	(CG)6	Exon	7	6	0.48
SSR19	*PH02Gene33375*	(CT)11	5′ UTR	3	3	0.16
SSR23	*PH02Gene44535*	(ACC)8	Exon	4	3	0.17
SSR32	*PH02Gene50471*	(CGG)5	Exon	2	2	0.03
SSR37	*PH02Gene27682*	(CT)7	Intron	12	9	0.81
SSR40	*PH02Gene44580*	(TGCA)4	Intron	2	2	0.08
SSR43	*PH02Gene19592*	(CGA)5	Exon	13	9	0.78
Mean				7.60	5.93	0.46

## Data Availability

Data are contained within the article.

## Supplementary Materials

The following supporting information can be downloaded at: https://www.mdpi.com/article/10.3390/genes13091518/s1. Table S1: List of 34 accessions used in this study; Table S2: The ten types of simple sequence repeats (SSRs) identified in five bamboo species; Table S3: Detail information of perfect SSRs identified in this study; Table S4: The SSR distribution in different regions of five bamboo species; Table S5: Detail information of primer sets designed in this study; Table S6: Peroxidase family members identified in five bamboo species according to Pfam annotation and Swiss-Prot annotation database; Table S7: The list of orthologous peroxidase gene pairs between *P. edulis* and other four bamboo species and paralogous peroxidase gene pairs in *P. edulis*; Table S8: Detail information of SSR identified in peroxidase genes of *P. edulis*; Table S9: Characteristics of 48 SSR markers used in this study; Figure S1: Twenty genotypes detected by SSR13; Figure S2: Thirteen genotypes detected by SSR43.
