# Supplementary figures and images for: Comprehensive Analyses of Simple Sequence Repeat (SSR) in Bamboo Genomes and Development of SSR Markers with Peroxidase Genes
Source: Genes (Basel). 2022 Aug 24;13(9):1518. doi: 10.3390/genes13091518 (PMC9498332; doi:10.3390/genes13091518)

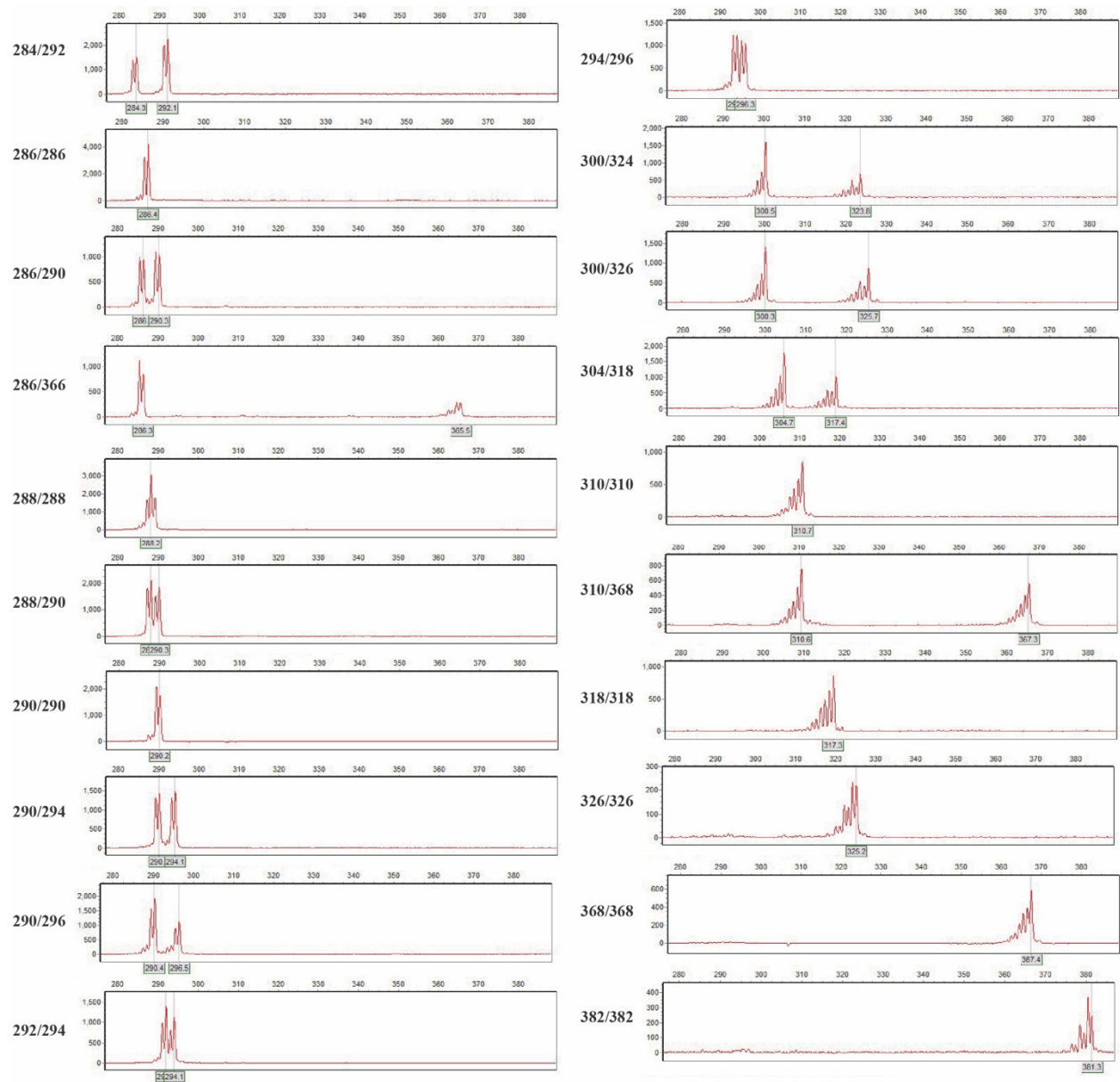

**Figure S1.** Twenty genotypes detected by SSR13.

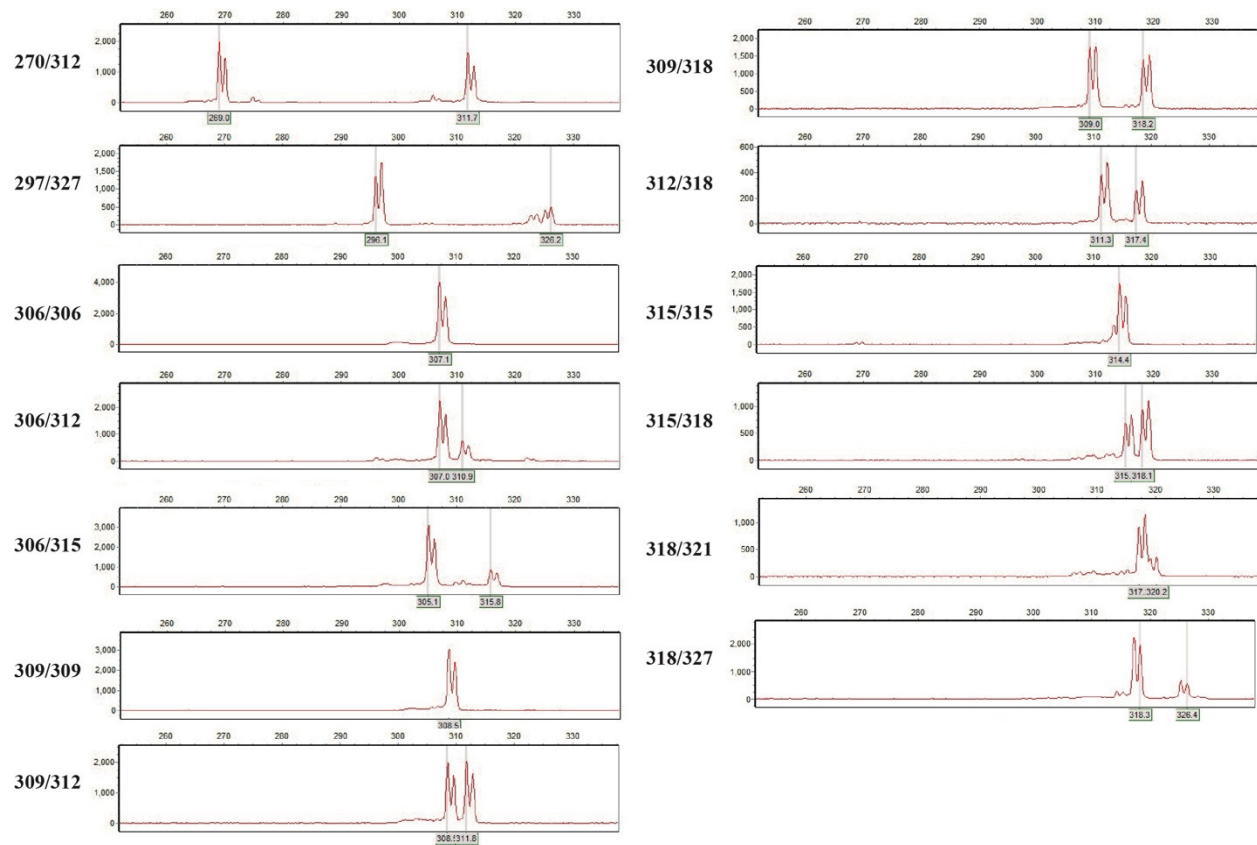

**Figure S2.** Thirteen genotypes detected by SSR43.

Supplement: Supplementary file 1 [file genes-13-01518-s001.zip › Supplementary Figure.pdf]
